# How to promote university technology transfer? A configuration analysis based on technology, organization and environment framework

**DOI:** 10.1371/journal.pone.0318563

**Published:** 2025-03-07

**Authors:** Xin Wang

**Affiliations:** College of Public Administration and Humanities, Dalian Maritime University, Dalian, China; Fooyin University, TAIWAN

## Abstract

University technology transfer (UTT) is at the forefront of innovation, representing the key to promoting the deep integration of science and technology with the economy. In order to explore mechanisms of realizing UTT, this study uses the fuzzy set qualitative comparative analysis (Fs-QCA) method to determine how the conditional configurations of technology, organization and environment (TOE) promote UTT. Evidently, there are four conditional configurations for promoting UTT, which are characterized by technology-organization-environment synergy, an organization-environment-orientation and a technology-organization-orientation. Technology transfer talents, organization construction, organization incentives, and market demand have an important influence on UTT. The main contribution of this study is its analysis of the nonlinear relationship between TOE conditions and UTT, which helps realize UTT in several ways.

## 1. Introduction

University technology transfer (UTT) can promote the precise connection between scientific and technological (S&T) supply and market demand, bring about tool revolution, efficiency revolution and industrial revolution, give birth to new industries, new models and new kinetic energy, and become the strategic cornerstone of enhancing S&T competitiveness and promoting new quality productive forces. Promoting S&T achievements in universities from laboratories to markets and promoting the transformation of R&D value into economic value are important steps for China to implement an innovation-driven development strategy, achieve a high-level of scientific and technological self-reliance and promote high-quality economic development. As important centers of S&T advancement, universities should actively transform S&T achievements into usable, productive and valuable technical products, so as to enhance the economic and social benefits of S&T achievements. At present, S&T achievements in universities are mostly produced in the form of “samples”, which usually do not meet the requirements of mass production; rather, the “sample” are the embryonic forms of “products” or “commodities”. According to the two-stage DEA model [1], the R&D and technology transfer activities of S&T achievements in universities mainly involve front-end (knowledge production) and middle-end (knowledge transformation) innovation. The outputs of front-end and middle-end innovation are resource inputs for back-end innovation, and front-end and middle-end innovation efficiencies directly affect back-end innovation.

In recent years, China has attached great importance to UTT. On one hand, the investment in UTT has increased rapidly. According to the *Compilation of Science and Technology Statistics of Colleges and Universities* published by the Ministry of Education in 2021, there were 44,749 full-time staff engaged in the application of R&D achievements and S&T services in Chinese universities, an increase of 73.13% compared with 2015. In 2021, the expenditure on applying R&D achievements and S&T service projects in Chinese universities was CNY 25,334,796 thousand, an increase of 84.02% compared with 2015. On the other hand, the outputs of UTT have also increased significantly with the continuous increase in investment in technology transfer. For example, in 2021, the number of technology transfer contracts, the amount of technology transfer contracts and the actual income from technology transfer in Chinese universities were 2.72 times, 2.53 times and 2.14 times greater than in 2015, respectively. However, the average price of UTT contracts in 2021 decreased by 7% compared with the average price in 2015. The price of S&T achievements in universities is affected not only by changes in the supply and demand relationship and market competition but also by technical content and added value. Therefore, the decline in UTT contract prices, to a certain extent, reflects lacking of quality and innovation value of S&T achievements in universities.

The above viewpoint is also supported by other scholars’ researches. Some research results show that the efficiency of UTT needs to be improved. Gao et al. [2] used a two-stage DEA model to analyze the technology transfer efficiency of 58 key universities directly under the Ministry of Education of China. The research showed that the efficiency of UTT in China was low and showed a downward trend. The main reason for this was that the efficiency of value creation was obviously lower than that of scientific research and innovation, which lowered the overall of technology transfer efficiency. In addition, the technology transfer efficiency of 985 universities was always lower than that of non-985 universities. Similarly, Yu et al. [3] divided the technology transfer process into two stages. The first stage was technology innovation, and the core task was to technologize knowledge innovation achievements. The second stage was value creation, and the core task was to commercialize technology innovation achievements. It was found that there were significant differences in the of technology transfer efficiency between the stage of technology innovation and the stage of value creation. According to Meng et al. [1], there were three reasons why UTT efficiency in China was low and it was difficult to meet the market demand. First, most of the scientific research achievements made at universities belonged to the field of basic research, and there was a certain lag in the value of basic research achievements. Second, the government continuously increased the funding for basic research in universities, while the market needed transformable and practical scientific research results, and the mismatch between supply and demand restricted UTT. Third, under the quantity-oriented evaluation system and transformation mode, Chinese universities were keen to produce a large number of low-quality scientific research achievements in a short amount of time, which were difficult to translate into economic and market value. Only high-quality scientific research achievements could meet the market demand, and further transformation was necessary.

Some research has discussed the linear relationship and net effect between UTT and its influencing factors. These influencing factors can be roughly divided into micro-internal and macro-external causes. For example, Pohlmann et al. [4] thought that UTT was related to organizational, technological, human and cultural factors including academic environment and industrial environment. Fang et al. [5] discussed the influence of government support, high-tech industry operation, financial institutions, technology intermediary, economic development, informatization level, and openness on UTT, and they analyzed the heterogeneous regional effects of these influencing factors. In addition, infrastructure construction represented by high-speed rail [6], social trust [7], and other factors, can strengthen industry-university cooperation, thus indirectly impacting UTT.

Other researchers opine that there is a nonlinear and asymmetric causal relationship between UTT and its influencing factors. UTT is the result of multiple influencing factors, and the permutation and combination of different factors have equivalent effects on UTT. Xie et al. [8] tried to break through organizational boundaries based on the perspective of multi-agent co-evolution and complementary advantages of the innovation ecosystem; analyzed the influence of the interaction among the government, market, universities and intermediary organizations on UTT; and explored paths to improve UTT efficiency under different regional conditions. Using the resource-based theory, Ma et al. [9] integrated the configuration analysis framework of university organizational resources and regional environmental resources affecting UTT and used a fuzzy set qualitative comparative analysis method to reveal the internal mechanism of multi-dimensional resource factors affecting UTT. Similarly, based on interface theory, Sun et al. [10] found the realistic paths to achieving UTT via multiple concurrent factors and complex causal mechanisms, including internal resource-driven, cooperation network resource-driven, internal comprehensive promotion, and external comprehensive promotion paths.

Configuration analysis helps reveal the realization mechanism and path of Chinese innovation in complex situations. China is determined to innovate in all aspects of technology, organization, market and policy, which has effectively promoted UTT. For example, in order to encourage UTT, China has pursued comprehensive innovation and reform experiments, reformed the mixed ownership of job-related S&T achievements, and increased the proportion of income from UTT used to reward scientific researchers. In order to strengthen the connection between industry and university, an order-based R&D and technology transfer mechanism of “targeted R&D, targeted transfer and targeted service” has been established, and an enterprise S&T commissioner system has been implemented. Through the establishment of the “1+3” service system comprising the technology trading market +  technology manager association, technology management companies and technology managers, the process of transferring technology from universities to the market has been accelerated.

Based on the above contexts, this research takes the TOE theoretical analysis framework as the basis for selecting influencing factors and uses the Fs-QCA method to analyze how various provinces and cities carry out “multiple solutions to one problem” with respect to promoting UTT.

The main ideas of this study are as follows: In Section 2, the connotation of UTT and its proxy indicators and influencing factors are reviewed and summarized, and the TOE theoretical analysis framework is introduced. In Section 3, the applicability of the fuzzy set qualitative comparative analysis method to this research is expounded, the selection basis and data sources of outcome and conditional variables are discussed, and the data calibration work is described. In Section 4, we analyze the necessity of using a single conditional UTT variable as the outcome variable and find a multivariate conditional configuration of UTT, and we test the robustness of the research results in various ways. In Section 5, combined with typical provincial and municipal cases, we discuss the practical significance and approaches of different conditional configurations, summarize our research conclusions, and put forward policy suggestions to promote UTT.

## 2. Literature review and theoretical framework

### 2.1. Measurement indicators of UTT

Regarding the statistical measurement of UTT, that is, how to measure UTT and what indicators express UTT, scholars have different opinions. Generally speaking, the statistical measurement indicators of UTT can be summarized in four types, which are absolute quantity indicators, relative quantity indicators, benefit indicators, and efficiency indicators. Some evidence from the literature is shown in Table 1.

**Table 1:** Measurement indicators of university technology transfer.

Types	Definition	Indicators
Absolute quantitative indicators	Used to measure the overall scale of S&T achievements, usually only involving the initial form.	Patent applications [11]
		Patent assignee changes [12]
		Research paper publications [13]
Relative quantitative indicators	Formed via the comparison of two absolute quantitative indicators related to S&T achievements. Used to measure the intensity, density, and prevalence of S&T achievements.	Number of actual valorization projects divided by number of potential valorization projects [14]
		Number of high-tech enterprises divided by number of higher education institutions [15]
		Number of academic patents sold divided by number of patents granted [16]
Benefit indicators	Used to measure the economic benefits generated by unit S&T achievements or the total economic benefits generated by all S&T achievements.	University licensing income; startup companies [17]
		Total number of technology transfer contracts and income [18]
		Technology transfer revenues [19]
Efficiency indicators	Used to measure the technology transfer output brought by unit technology transfer input.	The efficiency of university technology transfer [20]
		R&D expenditure divided by sales [21]
		Value added per employee [21]

As for the coverage of current indicators, the absolute quantitative indicators and relative quantitative indicators of UTT focus on measuring the form of expression S&T achievements, while some indicators only measure the initial forms of S&T achievements, such as publications, patents, and projects, failing to present the subsequent transfer and transformation process of S&T achievements. It is difficult to fully reflect the practical value and economic benefits of S&T achievements. Relatively speaking, the benefit and efficiency indicators of UTT highlight the economic value and market value of S&T achievements and show the dynamic process of transforming S&T achievements into real productivity. As for challenges in data collection, some research focuses on the micro-university level, and data mainly come from university websites, intellectual property offices, and third-party patent search platforms. Due to the differences in data scale and quality of search platforms, it will bring challenges to the authority and representativeness of data. Some research focuses on the macro-regional level, and data mainly come from statistical bulletins and statistical yearbooks. Different regions place differing emphasis on UTT; for example, some regions pay attention to technology input in universities, while others focus on technology output in universities, which will bring challenges to the statistical caliber of data.

### 2.2. Influencing factors of UTT

From the technology perspective, whether the S&T achievements of universities can be successfully transformed into real productivity depends, to a great extent, on the technical attributes of S&T achievements themselves. Barbolla et al. [22] thought that the lower the technical risk and the higher the technical feasibility, the more beneficial it was for UTT. When the main goal was to develop a new, usable product, the possibility of successful UTT was more probable. On the contrary, when the main goal was to integrate S&T achievements into more complex systems, the success rate of UTT decreased. Innovation resources are also an important factor influencing UTT. Battaglia et al. [23] combined grounded theory with the case study method and summed up the influencing factors that hindered UTT. Therefore, a lack of talent and a lack of financial support were regarded as resource obstacles to UTT.

From the organization perspective, UTT needs a stable organizational foundation and a favorable organizational environment, which can be physical, virtual, spiritual, or, more likely, all three [24]. As an organizational factor, organizational incentives [25] can enthuse R&D personnel, thus creating more transformable S&T achievements for universities. In an investigation study by Daniel et al. [26], 51% of inventors cited university incentives as their motivation to apply for patents. There are many forms of organizational incentives, among which money incentives and promotion incentives are common. Muscio et al. [27] found the influence of income distribution of technology transfer on UTT. Specifically, the withholding ratio of technology transfer income in universities significantly inhibited the growth of the number of university spin-offs, while the minimum share of equity held by scholars significantly promoted the growth of the number of university spin-offs. According to the research results of Ar et al. [28], the higher the credit of a patent application in academic promotion, the more beneficial it was to promote UTT.

From the environment perspective, Fang et al. [5] analyzed the role of multi-innovation subjects in UTT on the basis of the innovation ecosystem. The government was the policy maker and fund provider, universities and R&D institutions were the suppliers of knowledge and technology, industries were the fund providers, intermediaries were service providers, and financial institutions were investors. In addition, UTT is also affected by the market environment. Li et al. [29] divided the market environment into trust between universities and industries and the trading environment of the university technology market. Regarding the trust between universities and industries, universities were worried that the returns obtained from enterprises were not enough to support technology transfer activities, and industries were worried that the S&T achievements obtained from universities could not meet their manufacturing needs. Therefore, a lack of trust between universities and industries, to a certain extent, hindered UTT. As for the trading environment of the university technology market, the technology market provided an information exchange mechanism for universities as technology suppliers and industries as technology demanders and promoted S&T achievements from university laboratories to markets and from samples to commodities.

### 2.3. TOE framework

Tornatizky and Fleischer proposed the technology-organization-environment theoretical analysis framework (referred to as the TOE theoretical analysis framework), which is used to analyze the multi-dimensional factors influencing technological innovation diffusion [30]. Technical conditions mainly involve the attributes of technology itself, technological innovation [31], technological input and output [32], etc. Organizational conditions mainly involve organizational structure, resource endowment [30], organizational culture [33], etc. Environmental conditions mainly involve industrial demand [34], external support [35], market trends [36], etc. The TOE theoretical analysis framework is a systematic and integrated framework which comprehensively presents the internal and external causes of technological innovation diffusion from the micro-technical, meso-organizational, and macro-environmental levels. TOE, as a general theoretical analysis framework, allows researchers to flexibly set influencing factors and variables according to specific research problems and research situations [32] and has strong theoretical applicability. Therefore, TOE has been widely used by scholars for research topics such as digital economy development [37], low-carbon city governance performance [38], blockchain technology adoption and application [39], and artificial intelligence medical service resources application [40]. According to the research problems and realistic situations, researchers have provided a significant amount of empirical evidence for the TOE theoretical analysis framework and gradually expanded it from specific micro-organizational systems to complex and changeable macro-economic systems [41].

Technical conditions exert a micro-agglomeration effect on UTT. This is mainly reflected in the fact that universities gather innovative elements and resource endowments, such as funds, talents, and core technologies for technology transfer, and the process of gathering innovative elements and resource endowments for universities is the initial link to achieving UTT. According to the literature [32], this study divides the technology dimension into three conditional variables: technology transfer talents, technology transfer funds and technology innovation. First, UTT is a complex systematic project involving the front-end, middle-end and back-end of innovation. Every link needs a lot of financial support, and sufficient financial investment is an important condition for the smooth progression of UTT. Second, although researchers engaged in basic research have strong theoretical research abilities, most of their scientific research achievements are mainly completed in laboratories, and their adaptability to practical application scenarios and the external market environment is insufficient. Therefore, professionals who understand both technology and the market need to establish follow-up links to technology transfer. Thirdly, the innovation level of S&T achievements in universities determines the necessity of technology transfer and the market value of S&T achievements. The higher the technical content and innovation level of S&T achievements, the greater the economic benefits.

Organizational conditions exert a meso-allocation effect on UTT. This is mainly reflected in the rational allocation and utilization of innovative elements and resource endowments by means of organization and institutionalization. The allocation and utilization of innovative elements and resource endowments in universities is an intermediate link in UTT. In this study, the organizational dimension is divided into two conditional variables: organization construction [41] and organization motivation. R&D institutions are important platforms and places for UTT which provide the necessary funds, personnel, and scientific research equipment for UTT and also produce S&T achievements for technology transfer. In addition, incentive factors are a positive premise for influencing organizational innovation [42]. Improving the incentives for scientific research personnel is also an effective means of promoting UTT. In recent years, the comprehensive innovation reform experiment has been in full swing in China. An important measure is to deepen the reform of empowering S&T achievements in universities, and the inventors of S&T achievements can obtain technology transfer income generated by S&T achievements in proportion. Practice shows that similar measures to increase incentives for scientific research personnel arouse the enthusiasm of scientific research personnel to participate in technology transfer activities and promote UTT.

Environmental conditions exert a macro-leading effect on UTT. This is mainly reflected in the fact that UTT is not a process of “building a car behind closed doors”, but an open and innovative process of meeting the demand of the technology market and supporting the development of industry. Changes in supply and demand in the technology market and the industry development trend pave the way for UTT. According to the literature [43,44], this study divides the environmental dimension into two conditional variables: market demand and the industry-university relationship. On the one hand, the market plays a decisive role in resource allocation, and innovative resources usually flow to areas where demand exceeds supply and gradually tend to match supply and demand. In order to attract the agglomeration of innovative elements and enhance the economic value and market value of S&T achievements, UTT should not only face the forefront of world science and technology but also conform to the national development plan for industries, serving high-quality economic development and meeting the people’s need for a better life. On the other hand, due to the differences in the professional division of labor and innovation resources among different innovation subjects, technology transfer is a process of collaborative innovation among multiple subjects. The core advantages of universities lie in basic research and original innovation, but they lack the necessary technology and production chain for technology transfer, and the ability to obtain market information is insufficient. Compared with universities, the core advantages of enterprises and institutions are manufacturing, due to advanced production technology and perfect production lines, and strong sensitivity and responsiveness to the market. Therefore, the relationship between universities and enterprises and institutions is complementary in its advantages, and it is upstream and downstream in the process of technology innovation and technology transfer. Close cooperation between industry and universities is conducive to promoting UTT.

Under the traditional linear innovation model, scholars mainly pay attention to the single-directional influence of innovation activities. In the innovation ecosystem, scholars advocate a reciprocal and bidirectional relationship between science and industry [45,46]. As a part of the innovation ecosystem, there are also a reciprocal and bidirectional relationships among technology, organization, environment, and UTT. First, technology and UTT: The quality, level, and maturity of technology directly determine the feasibility and potential of UTT [47]. UTT can expand the application scenarios of technology, and the economic benefits generated can be further used for technology research and development [48]. Second, organization and UTT: Organizations can integrate and allocate innovation resources to create favorable conditions for UTT [49]. UTT can increase organizational income, enhance organizational operation ability, and motivate scientific researchers. Third, environment and UTT: The size of the market and industry demand. The intensity of competition will affect the direction and speed of UTT [50]. UTT can meet market demand, improve production equipment and techniques, and drive the transformation and upgrading of the supply chain industry [48]. Fourth, technology and organization: The development of technology can drive organizational changes, such as promoting R&D and technology transfer, promoting the flat development of R&D institutions and establishing new R&D institutions. Setting up specialized R&D institutions and teams and strengthening organizational incentives will engage scientific researchers in new technology R&D [51]. Fifth, technology and environment: The R&D of new technology can create new market demand and development opportunities, form new consumption growth points, and promote consumption upgrades [52]. Environmental factors will affect the R&D focus and application areas of the technology. Sixth, organization and environment: On the one hand, the organization exports talents and knowledge to the market and industry through S&T innovation, talent cultivation, social services, and other activities [53]. For example, the establishment and implementation of the S&T commissioner system is conducive to promoting the cooperation in industry - university research, improving the ability of universities to serve enterprises, and solving technical problems of enterprises. On the other hand, by carrying out joint R&D and entrusted R&D activities with universities, market subjects release clear market information for universities and promote the deep integration of S&T and the economy [53].

**Fig 1:**
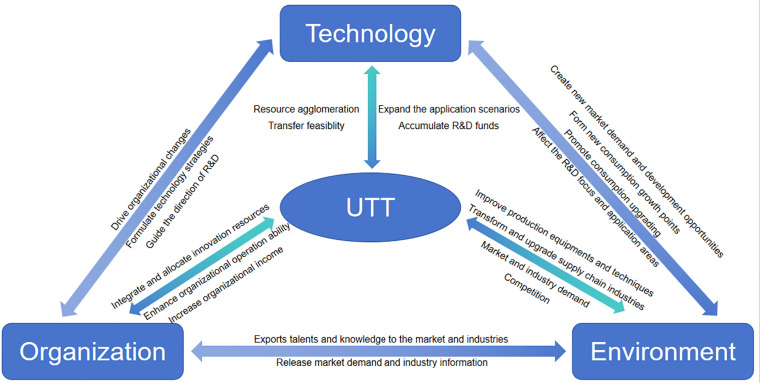
TOE theoretical analysis framework.

In conclusion, this research constructs the TOE theoretical analysis framework of UTT, as shown in Fig 1.

## 3. Research design

### 3.1. Methodology

Fuzzy set qualitative comparative analysis (Fs-QCA for short) is used to explore the nonlinear causal relationship between antecedents and results, which is characterized by “multiplicity” and “concurrency” [54]. “Concurrency” indicates that a single outcome can be influenced by multiple conditions simultaneously, highlighting the multifaceted nature of causality in social phenomena. “Multiplicity” refers to the presence of various configurations of antecedent conditions that can lead to the same outcome, illustrating that multiple pathways can result in similar effects [55], embodying the problem analysis principles of “multiple solutions to one problem” and “all roads lead to the same goal”. Unlike regression analysis, which focuses on the net effect of a single antecedent on the outcome, Fs-QCA is dedicated to discovering the comprehensive effect of multiple antecedents on the outcome. Fs-QCA combines quantitative and qualitative research effectively, making it possible to not only calculate the quantitative relationship between variables with the help of set theory and fuzzy mathematics methods, but to also explain the practical significance of the quantitative relationship by tracing back typical cases.

The TOE theory holds that S&T innovation and technology transfer are the results of the comprehensive influence of technical, organizational, and environmental antecedent conditions. Fs-QCA is used to analyze the effects of multiple antecedent conditions on outcomes and to reveal patterns in which different configurations of antecedent conditions lead to outcomes. Combined, the two imply that the causes of a certain result are not unique and aim to explore the comprehensive influence of antecedents on the result. The TOE theory can provide a theoretical basis and analytical framework for Fs-QCA, helping to identify key antecedent conditions affecting outcomes, while Fs-QCA can be used for empirical analysis to verify the relationships and hypotheses proposed in the TOE theory.

Additionally, this research meets the requirements of Fs-QCA in terms of sample size and variable attributes. Fs-QCA is suitable for the study of small- and medium-sized samples; this research takes 31 provinces in China as the research objects (Hong Kong, Macao and Taiwan are not included in the research scope due to data availability), and the sample size meets the recommended range of Fs-QCA. The data types of variables selected in this research are all continuous variables, which can be transformed into continuous fuzzy membership scores via Fs-QCA.

### 3.2. Variable selection and data source

Outcome variable (UTT): The knowledge and innovation generated by university researchers are transferred to industry through patent licensing, which leads to the commercialization of academic achievements [43]. Ravi et al. [56] believed that the practical feasibility of research achievements in universities was the core element for further transforming theoretical innovative ideas into new commercial products. The process of spreading innovation from universities and research institutes to other subjects with commercialization ability is called technology transfer. The process of technology transfer and commercialization in universities can be divided into seven stages, namely, scientific discovery, invention dissemination by R&D personnel, invention patent application evaluation, patent registration, technology marketing and supply, license negotiation, and formal or informal commercialization [57]. It can be seen that the foothold of UTT is commercialization and industrialization, and the main measure of commercialization and industrialization is sales revenue. Therefore, this research adopts the “actual income of technology transfer in universities” to express UTT.

Conditional variables: According to the TOE theoretical analysis framework, this research holds that the outcome variable of UTT is comprehensively influenced by three dimensions of conditional variables: technology, organization and environment. Therefore, the technology dimension consists of technology transfer funds, technology transfer talents, and technology innovation. The organization dimension consists of organization construction and organization incentives. The environment dimension consists of industry-university relations and market demand.

Technology transfer talents: Due to limited cognitive ability, time, and energy, it is difficult for researchers to attend to scientific research and technology transfer at the same time, so it is necessary for specialized technology transfer talents to conduct the transformation of S&T achievements. In the *Opinions on Deepening the Reform of Project Evaluation, Talent Evaluation and Institutional Evaluation* issued by the General Office of the CPC Central Committee and the General Office of the State Council, the basic principles of talent classification evaluation are proposed, and the personnel engaged in applied technology development and achievement transformation are categorized separately, and the evaluation indicators are proposed, which shows the importance of technology transfer talents. Full-time personnel engaged in applying R&D achievements and S&T services (including scientific research management) account for 90% or more of their total working hours. Referring to reference [58], this research uses “full-time personnel of R&D achievements application and S&T service” to express technology transfer talents.

Technology transfer funds: UTT is a high-investment, high-risk and high-income activity. A large amount of funds are needed for the purchase of technology transfer equipment, the introduction and retention of technology transfer talents, the establishment of a technology transfer service platform, and marketing and publicizing S&T achievements. In this research, “expenditure on R&D achievements application and S&T service projects” is used to express technology transfer funds.

Technological innovation: Patent applications and authorizations are usually used to indicate the technology innovation capability of a region or organization. A patent application means that an innovation subject has performed innovation activities, made S&T achievements, and applied for legal protection. Patent authorization shows that the innovation value of these S&T achievements has been recognized by government departments and protected by intellectual property law, patent law, and other laws. Compared with design and utility model patents, invention patents are difficult to develop and have high innovation value. Referring to reference [15,58], this study uses “the number of invention patents granted” to express technology innovation.

Organization construction: Adequate technical reserves and abundant S&T achievements are prerequisites for UTT. The construction of R&D institutions is an effective way to create new technologies and realize new achievements. The construction of R&D institutions can promote the agglomeration of innovative resources, accelerate the accumulation of technology, innovate S&T achievements, and improve scientific research strength overall. In recent years, universities have actively cooperated with the government, enterprises, and national scientific research institutions to establish new R&D institutions, aiming at research, development and transformation, reflecting a new type of industry-university research partnership, which is conducive to promoting technology transfer [59]. In this research, “the number of R&D institutions” is used to express organization construction.

Organization incentives: R&D personnel are producers of intellectual property rights. In order for R&D personnel to create intellectual property rights for an organization, the organization should have a reward system to encourage R&D personnel to create intellectual property rights [60]. Scientific research personnel fees refer to the expenses used to pay wages, allowances, bonuses, and other forms of labor remuneration to researchers, technicians, and auxiliary personnel participating in scientific research projects. The increase in scientific research personnel fees in universities is conducive to stimulating the initiative of scientific research personnel in universities to carry out technology innovation and technology transfer. In this research, “the proportion of scientific research personnel expenditure to internal expenditure of S&T funds” is used to express organization incentives.

Industry–university relations: Industry–university cooperation is an effective way to enhance the value of academic achievements and patent applications. Industry–university cooperation can combine the resource advantages of existing equipment, technology, and R&D personnel in universities with industrial needs and marry basic research and applied research, thus generating economic benefits and improving national innovation ability [61]. Industry–university relations are an important indicator to measure collaborative innovation. According to the 2023 Global Innovation Index Report, the sub-index of China’s industry–university research cooperation ranks sixth in the world. According to the literature [15], this research adopts “the proportion of entrusted funds of enterprises and institutions to S&T funds” to express the industry–university relations.

Market demand: The technology market is a platform for technology trade between technology suppliers and technology demanders, and it is also an important channel for universities’ S&T achievements to progress from innovation benefits into economic and social benefits. The technology market releases clear transaction information to innovation subjects, such as universities, which can dispel the information asymmetry among innovation subjects, to a certain extent, and help universities carry out technology transfer work more pertinently. In this research, “technology market turnover” is used to express market demand.

In summary, the variable system of this research is shown in Table 2.

**Table 2:** Variable system.

**Types**	**Dimensions**	**Names**	**Indicators**	**Abbreviations**
Outcome	Technology transfer in universities		Actual income from technology transfer	UTT
Conditions	Technology	Technology transfer talents	Full-time personnel of R&D achievements application and S&T service	FTS
		Technology transfer funds	Expenditure of R&D achievements application and S&T service projects	EXP
		Technology innovation	Number of invention patents granted	PAT
	Organization	Organization construction	Number of R&D institutions	INS
		Organization incentives	Proportion of scientific research personnel expenditure to internal expenditure of S&T funds	ORI
	Environment	Industry–university relations	Proportion of entrusted funds of enterprises and institutions to S&T funds	IUR
		Market demand	Technology market turnover	TMT

The research data come from the *Compilation of Science and Technology Statistics in Colleges and Universities*, *China Statistical Yearbook,* and *China Science and Technology Statistical Yearbook*. Number of universities in different regions is shown in Table 3. As for the time lags for transferring scientific discoveries from universities to industry, Anderson et al. [17] included preceding years as separate inputs to allow for separate time lags between research expenditures and the UTT outcomes. It is found that UTT outcomes are robust with respect to the inclusion of highly correlated inputs. This means that due to the high correlation and strong substitution of research expenditures in different years, their impacts on UTT outcomes would be very similar. Refering to reference [5], and considering that the influence of conditional variables on outcome variables may lag behind, the data from 2020 is used for conditional variables and 2021 is used for outcome variables.

**Table 3:** Number of universities.

Region	Number of univesities	Region	Number of universities
Beijing	47	Hubei	85
Tianjin	16	Hunan	98
Hebei	110	Guangdong	118
Shanxi	70	Guangxi	63
Inner Mongolia	41	Hainan	15
Liaoning	49	Chongqing	60
Jilin	54	Sichuan	115
Heilongjiang	39	Guizhou	60
Shanghai	34	Yunnan	67
Jiangsu	149	Tibet	4
Zhejiang	92	Shaanxi	74
Anhui	98	Gansu	43
Fujian	86	Qinghai	11
Jiangxi	85	Ningxia	11
Shandong	120	Xinjiang	34
Henan	143	N/A	N/A

### 3.3. Data calibration

Calibration is the process of transforming distance scale variables into fuzzy membership scores. Referring to the research experience of Kou et al. [62], this research adopts a direct calibration method and sets 95%, 50%, and 5% quantiles of the original data of outcome variables and conditional variables as complete membership, crossover, and complete non-membership data calibration anchor points, as shown in Table 4.

**Table 4:** Calibration anchor points.

Variable type	Variable name	Complete membership	Crossover	Complete non-membership
Outcome	UTT	513825	59442	1001
Conditions	FTS	3446.5	942	51
	EXP	2680043.5	388555	28329
	PAT	12860.5	2372	79
	INS	888	384	43.5
	ORI	0.325	0.211	0.117
	IUR	0.325	0.211	0.066
	TMT	26775305	3797786	128374.5

## 4. Qualitative comparative analysis

### 4.1. Necessary condition analysis

Necessary condition analysis is used to discuss the extent to which the set of outcome variables constitutes a subset of the set of conditional variables. Consistency is an important indicator for judging whether a certain condition can become a necessary condition. When the consistency is higher than 0.9, the condition is considered a necessary condition [63], which means that the condition always exists in a certain outcome. The results of the necessary condition analysis are shown in Table 5, and the consistency of all conditional variables is less than 0.9, which shows that the single conditional variables of technology, organization, and environment are not enough to promote UTT, and UTT is the result of the comprehensive action of the three.

**Table 5:** Analysis of necessary conditions.

Conditions	Consistency	Coverage
FTS	0.872	0.880
~FTS	0.449	0.388
EXP	0.805	0.886
~EXP	0.507	0.409
INS	0.871	0.828
~INS	0.408	0.372
PAT	0.875	0.920
~PAT	0.469	0.392
ORI	0.712	0.684
~ORI	0.510	0.461
IUR	0.799	0.752
~IUR	0.501	0.462
TMT	0.834	0.879
~TMT	0.473	0.395

“~” means that the conditional variable does not appear, the same below.

### 4.2. Conditional configuration analysis

Since all the conditional variables under the TOE framework are not conditions necessary to promote UTT, which combination of conditional variables can promote UTT? Conditional configuration analysis is used to explore the comprehensive effects of technology, organization, and environment on UTT. In a qualitative comparative analysis, there are parsimonious solutions, intermediate solutions, and complex solutions. Because the intermediate solution can better combine theory with practical cases than the parsimonious solution and complex solution and have more practical significance, this research adopts the conditional configurations generated by the intermediate solution and identifies the core conditions and periphery conditions in each conditional configuration with a parsimonious solution to explain the multiple concurrent causal relationships between the combination of conditional variables at the technical, organizational, and environmental levels and UTT. The calculation results are shown in Table 6. Therefore, the core conditions refer to the conditional variables existing in the intermediate solution and the parsimonious solution at the same time, which has an important influence on the outcome variable. Periphery conditions are deleted in the parsimonious solution and only exist in the intermediate solution, which plays an auxiliary role in the outcome variable [64].

**Table 6:** Analysis of conditional configurations.

Conditions Configurations	C1	C2	C3	C4
FTS	·	ο	●	●
EXP	·	ο	ο	·
PAT	·		ο	·
INS	●	●	ο	ο
ORI		·	●	●
IUR		ο	·	·
TMT	●	●	ο	ο
Raw coverage	0.672	0.256	0.268	0.254
Unique coverage	0.438	0.035	0.020	0.013
Consistency	0.976	0.953	0.965	0.992
Solution coverage	0.798			
Solution consistency	0.959			

“●” means that the core condition variable appears, “O” means that the core condition variable does not appear, “·” means that the periphery condition variable appears, “ο” means that the periphery condition variable does not appear, and a blank space means that whether the condition variable appears has no effect on the outcome variable; the same is true below.

The overall consistency of the intermediate solution is 0.959, which shows that 95.9% of the provincial and municipal universities have higher technology transfer levels in the cases that meet these four conditional configurations. The overall coverage of the intermediate solution is 0.798, which shows that these four conditional configurations can explain 79.8% of the cases with a high-level of UTT. Generally speaking, the four conditional configurations generated by the intermediate solution have strong explanatory power.

Configuration C1: FTS*EXP*PAT*INS*TMT→UTT (“*” means “and”, “→” means “trigger”, bold characters mean core conditions, the same below). This configuration shows that UTT can be promoted by taking widely established R&D institutions and active technology markets as the core conditions and taking sufficient technology transfer funds, a large number of technology transfer talents and solid technology reserves as the periphery conditions. This configuration comprehensively covers the conditional variables of technology, organization, and environment, and the provincial and municipal cases that meet this configuration often have rich internal resource endowments and superior external development environment. It reveals the interactive mechanism of technology, organization, and environment of UTT. Universities strengthen the gathering of technology transfer funds and human resources, accelerate the accumulation of technology, and provide technical element support for the construction of specialized R&D institutions. R&D institutions constantly optimize the allocation of technology transfer resources to adapt the direction and speed of technology transfer to the development of the technology market, thus promoting the transformation of technological benefits into economic benefits.

Configuration C2: ~ FTS*~EXP*INS*ORI*~IUR*TMT→UTT. This configuration shows that in the case of great demand for the technology market, if universities can pay attention to the construction of R&D institutions and increase the incentives for scientific research personnel, the problems caused by insufficient investment in technology transfer funds and talents and less contact between industry and university can be overcome, thus promoting UTT. This configuration mainly seeks a solution for UTT from the level of organizational construction and organizational incentives. It reveals the interactive mechanism between the organization and the UTT environment. The technology market is the demand side of technology, while universities are the suppliers of technology. Changes in the external market environment can guide the internal organizational planning and layout of universities. The stronger the demand for a certain technical field in the technology market, the more universities are inclined to strengthen the construction of R&D institutions and incentives for scientific researchers in this technical field so as to promote the matching of technology supply and demand.

Configuration C3: FTS*~EXP*~PAT*~INS*ORI*IUR*~TMT→UTT. This configuration indicates that under circumstances in which there is less investment in technology transfer funds, weak technology innovation ability, few R&D institutions, and insufficient technology market demand, universities should adhere to the talent-driven orientation, continuously increase investment in and the incentives of technology transfer talents, and strengthen relations between industry and universities to promote UTT. The input and incentives of technology transfer talents are the core conditions of this configuration, and the relations between industry and university are a peripheral condition. This is a talent-oriented approach to achieving high UTT performance, which also reveals the interactive mechanism between technology and the organization of UTT. On the one hand, technology transfer talents in universities are necessary organizational elements. On the other hand, universities strengthen organizational incentives to attract increments and revitalize stocks of technology transfer talents to participate in industry – university cooperation. Core technological and organizational conditions complement each other to jointly promote UTT.

Configuration C4: FTS*EXP*PAT*~INS*ORI*IUR*~TMT→UTT. This configuration indicates that when universities have a strong technology innovation ability, large investment in technology transfer funds and talents, strong organization incentives, and close relations between industry and university, technology transfer can be realized even if the number of R&D institutions is small and the technology market is not active enough. From the perspective of core conditions, it is also a talent-oriented mechanism of achieving high UTT performance. Compared with configuration C3, configuration C4 reveals the auxiliary influence of technology transfer funds and technology innovation. On one hand, by strengthening technological innovation, universities can produce more high-quality S&T achievements and enhance the source supply capacity of technology transfer. On the other hand, universities guide technology transfer talents to transform S&T achievements into real productive forces through industry–university cooperation by increasing technology transfer funding and organizational incentives.

### 4.3. Robustness test

This research adopts two ways to test robustness. First, it adjusts the consistency threshold [65]. Based on the experience of Yin et al. [66], this research raises the consistency threshold from 0.80 to 0.85, and the results of the conditional configuration analysis were consistent with those shown in Table 6. Second, it resets the anchor point of data calibration [67]. In this research, the 90%, 50%, and 10% quantiles of the original data of the outcome variable and conditional variables are set as the data calibration anchor points with complete membership, crossover, and complete non-membership, and the conditional configuration analysis is carried out again. The intermediate solution generates four condition configurations, as shown in Table 7. From the fitting parameters, the overall coverage and overall consistency of the newly obtained intermediate solution are 0.808 and 0.960, respectively, which are higher than those in Table 6. From the point of view of conditional configuration, the newly obtained intermediate solution is composed of four conditional configurations, and the distribution of conditional variables in each configuration and the actual case situation that can be explained are highly consistent with the results in Table 6. Therefore, after repeated tests of the two methods, this research has obtained consistent research results, and the research results have strong robustness.

**Table 7:** Robustness test results.

Conditions Configurations	C1	C2	C3	C4
FTS	·	ο	●	●
EXP	·	ο	ο	·
PAT	·		ο	·
INS	●	●	ο	ο
ORI		·	●	●
IUR		ο	·	·
TMT	●	●	ο	ο
Raw coverage	0.686	0.176	0.199	0.196
Unique coverage	0.522	0.037	0.020	0.023
Consistency	0.979	0.936	0.968	1
Solution coverage	0.808			
Solution consistency	0.960			

## 5. Discussion and conclusions

### 5.1. Typical cases of conditional configurations

Due to the great differences in the technological, organizational and environmental conditions, different provinces and municipalities have different ways of promoting UTT. The supporting cases for each conditional configuration are shown in Table 8.

**Table 8:** Supporting cases of conditional configurations.

Configurations	C1: FTS*EXP*PAT*INS*TMT→UTT	C2: ~FTS*~EXP*INS*ORI*~IUR*TMT→UTT	C3: FTS*~EXP*~PAT*~INS*ORI*IUR*~TMT→UTT	C4: FTS*EXP*PAT*~INS*ORI*IUR*~TMT→UTT
Supporting cases	Jiangsu Beijing Hubei Guangdong Shaanxi Zhejiang Shandong Hunan Liaoning	Anhui Jilin	Jiangxi	Chongqing

In terms of efficiency and effectiveness, the labor productivity of all technology transfer employees (the ratio of the actual technology transfer income to the full-time personnel applying R&D achievements and S&T service, UTT/FTS) in these regions is slightly different. The labor productivity of all technology transfer employees in some regions is relatively high. For example, it is CNY 291.95 thousand/person in Beijing, CNY 165.35 thousand/person in Guangdong, and CNY 150.84 thousand/person in Zhejiang, indicating that universities in these regions mainly promote technology transfer by improving efficiency. While the labor productivity of all technology transfer employees in some regions is relatively low, for example, it is CNY 77.05 thousand/person in Jiangsu, CNY 73.7 thousand/person in Shandong, and CNY 60.31 thousand/person in Jiangxi, which indicates that universities in these regions mainly promote technology transfer by increasing the investment scale of technology transfer resources.

Cases that meet the criteria of configuration C1 include Jiangsu, Beijing, Hubei, Guangdong, Shaanxi, Zhejiang, Shandong, Hunan, and Liaoning. Most of these provinces and municipalities are located on the eastern coast or are pilot zones for comprehensive innovation and reform, with superior geographical positions, abundant innovation resources, high synergy between economic construction and social development, perfect innovation systems and sound systems and mechanisms. Taking Jiangsu as an example, Jiangsu is located in the Yangtze River Delta region, with superior location conditions. Local universities have a strong siphon effect on innovative resources, attracting innovative resources to gather at local universities. On the technical level, in 2021, the expenditure of applying R&D achievements and S&T service projects, full-time personnel applying R&D achievements and S&T services, and the number of invention patents granted in Jiangsu universities ranked in the top two in China. At the organizational level, Jiangsu formulates and implements policies and measures to promote the construction of innovation consortia and unites universities and small- and medium-sized enterprises upstream and downstream of the industrial chain to take the lead in building a number of task-based innovation consortia with common interests as the link and market mechanism as the guarantee. On the environmental level, Jiangsu has perfected and applied new modes and mechanisms of technology transfer services, such as “taking the lead in the list” and publicizing and listing S&T achievements. In order to surpass the “last mile” of technology transfer, Jiangsu vigorously cultivates a team of technical managers. Rich internal resource endowment and a superior external development environment provide powerful endogenous and exogenous power for promoting UTT.

Cases that meet configuration C2 include Anhui and Jilin. Although Anhui is located in the Yangtze River Delta region, it is located at the edge of the innovation niche in the Yangtze River Delta region. Affected by the siphon effects of the core and sub-core of the Yangtze River Delta region, it is difficult for innovation resources to gather in Anhui. Jilin is located in Northeast China. Affected by the overall economic situation and business environment in Northeast China, it is difficult to generate innovation resources incrementally. Therefore, in the case of lacking incremental innovation resources, Anhui and Jilin focus on revitalizing the stock of innovation resources to promote UTT. For example, Anhui, as a pilot zone of comprehensive innovation and reform, has set the minimum standard of the proportion of technology transfer rewards to technology transfer income at 50% in order to increase the incentives for scientific research personnel to engage in technology transfer. On this basis, universities and research institutes are allowed to set specific proportions independently, and technology transfer rewards are not included in the total performance pay.

The case conforming to configuration C3 is Jiangxi. On one hand, Jiangxi adheres to the talent drive and constantly increases the investment and incentives for technology transfer talents in universities. For universities with outstanding achievements, such as achievement transformation and social services, the human securities and financial departments should give appropriate inclination when verifying the total performance pay, and the inclined part is mainly used for the distribution of incentive pay for scientific research personnel. Universities should be given autonomy in distribution. Universities can formulate assessment, distribution, and reward methods that reflect their own characteristics according to their actual situation and adopt flexible and diverse distribution methods to distribute independently within the approved total performance pay, focusing on key positions, business backbones, and scientific research personnel who have made outstanding achievements. On the other hand, Jiangxi continues to strengthen the relations between industry and the university. Jiangxi vigorously promotes the construction of “one industry, one university”, strategic cooperation between strategic emerging industries and advantageous industries, and universities at home and abroad and help optimize industries. Jiangxi promotes the construction of industrial technology research institutes. Jiangxi encourages development zones at or above the provincial level to take the lead, focusing on their leading industries, relying on key enterprises in development zones to unite universities and research institutes, and jointly set up industrial technology R&D and innovation entities to break through industrial technology bottlenecks. Jiangxi implements a policy of developing and transforming new technologies and products. Moreover, Jiangxi encourages small- and medium-sized enterprises to cooperate with universities to develop new technologies and products, and accelerate the transformation of S&T achievements.

The case that exemplifies configuration C4 is Chongqing. First, Chongqing attaches great importance to the cultivation and incentives of technology transfer talents in universities. Chongqing supports universities to add technical brokerage positions in the teaching and research team, implement categorizing management for the evaluation and employment of professional titles of technical brokerage, and take the transforming effect of the S&T achievements of university teachers as an important indicator of the evaluation and employment of professional titles. Individuals and teams should be rewarded according to the technology transfer performance level. Chongqing deepens the reform of empowering S&T achievements in universities. Secondly, Chongqing attaches importance to the transfer and transformation of high-value patents in universities. Chongqing supports qualified universities to sort out and formulate lists of convertible high-value patents, promote the cultivation and transformation of high-value patents, and build a high-value patent industry–university research operation consortium. Third, Chongqing pays attention to promoting the docking of S&T achievements in universities and deepening the cooperation between industry and universities, such as improving the digital intelligence service of S&T achievements in universities, optimizing the docking mechanism of normalized roadshows, promoting the construction of innovation and an entrepreneurship ecological circle around universities, and establishing major intellectual property operation companies and one-stop transfer and transformation service platforms.

### 5.2. Implications

As for theoretical implications, although current research has deeply analyzed the influencing factors and mechanisms of UTT and approaches to realizing UTT, there some research questions remain to be discussed. Current research, on the factors influencing UTT is mainly based on the linear hypothesis and the idea of controlling variables, exploring the net effect of specific core variables on UTT [4–7], but it pays less attention to the comprehensive effect of various factors on UTT. On the basis of the nonlinear hypothesis and TOE framework, this research holds the opinion that a single factor is not sufficient to promote UTT, selects some of the variables that are proven to trigger UTT in the current literature, and discusses the “multiplicity” and “concurrency” of various factors that result in UTT. This research discovers four equivalent conditional configurations for promoting UTT.

As for practical implications, we should carry out policy innovation according to local conditions, proposing differentiated and diversified policy instrument combinations that are conducive to promoting UTT and serve as a boxing of policy combinations. When formulating policies, we should not treat technological, organizational, and environmental conditions in isolation but strengthen the synergistic coupling relationship among the three. In addition, we should adhere to the problem orientation, summarizing the advantages and disadvantages of regional universities in technology, organization, and environment, adjusting their positioning, fostering strengths and circumventing weaknesses, and adopting a dislocation development path.

Universities should combine various conditions to achieve UTT when they lack specific technological, organizational, or environmental conditions. The choice of combining conditions is a response to compensating for deficiencies. For example, when technical conditions are lacking, universities should take the external technology market demand as a guide, coordinate organizational resources, establish market-oriented R&D institutions, increase organizational incentives, raise the minimum standard of the proportion of technology transfer rewards to technology transfer income, and attract technology transfer resources to gather in universities. When the external technology market is sluggish, universities should strengthen their investment in R&D and technology transfer; unblock the technology transfer channels among basic research, applied research, and experimental development research; rely on key enterprises to jointly set up industrial R&D innovation entities; establish industry–university cooperation; promote consumption upgrading and industrial upgrading with technological (CSV)upgrading; and form new market demand.

## 6. Conclusions

First, the single effects of technology, organization and environment alone are not enough to promote UTT. In other words, UTT is the result of the comprehensive effect of technology, organization and environment.

Secondly, there are four conditional configurations of UTT, which can be further summarized into three types, namely, which are characterized by TOE synergy (C1), an OE-orientation (C2) and a TO orientation (C3, C4). Each condition configuration is a differentiated permutation and combination of technological, organizational, and environmental conditions, which embodies the concept of “all roads lead to the same goal”. There are various solutions to UTT.

Thirdly, technology transfer talents, organization construction, organization incentives, and market demand are important conditional variables for promoting UTT. They are core conditions that appear in different conditional configurations. Therefore, it is suggested that universities should take market demand as their guiding orientation, focus on introducing and providing incentives to technology transfer talents, and actively establish R&D institutions.

## Supporting information

S1 File Raw data.
(CSV)
